# Indirect quantification of mitral regurgitation using cardiovascular magnetic resonance: A comparison of techniques

**DOI:** 10.1186/1532-429X-17-S1-P361

**Published:** 2015-02-03

**Authors:** Christian L Polte, Odd Bech-Hanssen, Åse A Johnsson, Sinsia A Gao, Kerstin M Lagerstrand

**Affiliations:** 1 Cardiology, Sahlgrenska University Hospital, Gothenburg, Sweden; 2 Clinical Physiology, Sahlgrenska University Hospital, Gothenburg, Sweden; 3 Radiology, Sahlgrenska University Hospital, Gothenburg, Sweden; 4 Diagnostic Radiation Physics, Sahlgrenska University Hospital, Gothenburg, Sweden

## Background

Indirect quantification of mitral regurgitation (MR) by cardiovascular magnetic resonance (CMR), currently used as a second-line diagnostic tool after echocardiography, can be achieved by three methods. Each method has its advantages and disadvantages due to the limitations of the underlying techniques used to determine ventricular volumes and/or flow information. The aim of the study was to compare all three indirect CMR methods to determine their agreement in grading MR severity.

## Methods

This prospective study comprised 29 patients (mean age 60 ± 11 (standard deviation (SD)), 97% males) with severe chronic MR who underwent an echocardiographic and CMR exam prior to surgery. Steady-state free precession sequences were used to obtain a short-axis data set to evaluate left and right ventricular stroke volumes (LV/RVSV) and phase-contrast velocity sequences were applied to obtain aortic forward flow (AoFF) and mitral inflow (MiIF). The mitral regurgitant volume (MRV) was determined by the ‘standard' (MRV = LVSV - AoFF), ‘volumetric' (can only be used in the absence of multivalvular disease and intra-cardiac shunt; MRV = LVSV - RVSV) and ‘flow' method (MRV = MiIF - AoFF). The mitral regurgitant fraction (MRF) was calculated as follows: MRV/LVSV x 100% (‘standard'/‘volumetric' method) or MRV/MiIF x 100% (‘flow' method). Agreement between the techniques was evaluated using the Bland-Altman method.

## Results

The main indication for surgery was symptomatic MR (n=27) or severe LV dilatation. All patients had a dilated LV (mean end-diastolic volume 281 ± 49 ml) with preserved systolic function (mean ejection fraction 64 ± 5 %) secondary to degenerative mitral valve disease with prolapse (Carpentier type II). Seven patients had ≥ mild pulmonary and/or tricuspid regurgitation according to echocardiography and were therefore excluded from the ‘volumetric' method. All three techniques determined an overall significant different MRV and MRF (Table 1). There was good to moderate agreement between the methods despite wide 95% limits of agreement (Table 2). The MRV was below the guideline cut-off value of 60 ml in 0%, 14% and 17% of the cases, and the MRF was below the guideline cut-off value of 50% in 24%, 68% and 52% of the cases, using the ‘standard', ‘volumetric' and ‘flow' method respectively.

**Table 1:** Comparison of the indirect CMR methods for MR quantification.

					Post-hoc analysis		
	‘Standard' method	‘Volumetric' method	‘Flow' method	Overall P-value	‘Standard' versus ‘volumetric' method	‘Standard' versus ‘flow' method	‘Volumetric' versus ‘flow' method

MRV (ml)	100 ± 25	85 ± 27	82 ± 23	< 0.0001	< 0.0001	< 0.0001	0.37

MRF (%)	55 ± 7	45 ± 9	50 ± 9	< 0.0001	< 0.0001	< 0.0001	0.08

Data are presented as the mean ± standard deviation (SD). The significance of the differences between the different methods is presented as P-values. CMR, cardiovascular magnetic resonance; MR, mitral regurgitation; MRF, mitral regurgitant fraction; MRV, mitral regurgitant volume

**Table 2:** Bland-Altman analysis of the indirect CMR methods for MR quantification.

	‘Standard' versus ‘volumetric' method	‘Standard' versus ‘flow' method	‘Volumetric' versus ‘flow' method
Mitral regurgitant volume			

r	0.92	0.87	0.85

MD ± SD (ml)	17 ± 12	17 ± 18	3 ± 20

LoA (ml)	-7 to 41	-18 to 52	-36 to 42

Mitral regurgitant fraction			

r	0.76	0.80	0.58

MD ± SD (%)	9 ± 6	5 ± 5	-4 ± 9

LoA (%)	-3 to 21	-5 to 15	-22 to 14

The difference between the methods is presented as the correlation coefficient (r), mean difference (MD) ± SD and 95% limits of agreement (LoA). Otherwise abbreviations as in Table 1

## Conclusions

In the present study, we demonstrate that the three indirect CMR methods for MR quantification show good to moderate agreement although each technique determines an overall significant different MRV and MRF. In clinical practice, the choice of method might therefore affect the grading of MR severity and thereby the timing of surgery. Due to the lack of a true "gold standard", it is not possible to say which method quantifies MR severity with the highest accuracy and reliability.

## Funding

This study was funded by a project grant from the Health & Medical Care Committee of the Regional Executive Board, Västra Götaland Region, Sweden.

